# Locally obtained autologous bone grafts are effective for achieving arthrodesis while managing foot and ankle charcot’s neuroarthropathy: short to mid-term results from a specialized north African foot and ankle surgery unit

**DOI:** 10.1186/s13018-024-05036-9

**Published:** 2024-09-16

**Authors:** Ahmed Ekram Osman, Wael EL-Adly, Kerolos Maged Haroun, Mohamed Khaled, Ahmed A. Khalifa

**Affiliations:** 1 Orthopaedic Department, Assiut University Hospital, Assiut, Egypt; 2 Orthopaedic Department, Qena faculty of medicine and University Hospital, South Valley University, Qena, Egypt

**Keywords:** Charcot’s neuroarthropathy, Arthrodesis, Autograft, Limb-salvage

## Abstract

**Purpose:**

We aimed to report the union rate after only utilizing a locally obtained autologous bone graft while correcting the deformity and performing joint arthrodesis in patients with foot and ankle Charcot neuropathy (CN) and to report on the radiographic, functional, complications incidence outcomes at a minimum of two years of follow up.

**Methods:**

We included 24 patients having a mean age of 55.4 ± 10.1 years diagnosed with CN of the foot, ankle, or both. Seven (29.2%) cases were classified as Brodsky type 1, 11 (45.8%) as type 3 A, and six (25%) were type 4. Hindfoot and Midfoot bi-columnar arthrodesis was performed in 70.8% and 29.2% of the patients, respectively. Eight (33.3%) cases had preoperative ulcers. Functional outcomes were evaluated using a modified AOFAS score. Arthrodesis site union was assessed clinically and radiographically. All patients were available for a mean follow up of 35.7 ± 9.5 (24–54) months.

**Results:**

Arthrodesis site union was achieved in 23 (95.8%) cases after a mean of 4 ± 1.7 (2-7.5) months. The mean modified AOFAS score was 72.4 ± 10.41 (46–83) points; 79.2% achieved excellent and good scores. Ulcers healed in 87.5% of the patients. Twenty-two (91.7%) patients were satisfied with their functional results. Infection incidence was 12.5%, and no patients required revision or amputation.

**Conclusion:**

Foot and ankle Charcot neuroarthropathy deformity correction by arthrodesis of the affected joint as a salvage management option resulted in acceptable clinical and radiological outcomes. To enhance the local environment for arthrodesis consolidation, locally obtained autografts led to higher union rates and avoided the drawbacks of using other graft types.

## Introduction

Foot and ankle Charcot neuroarthropathy (CN) is a devastating condition affecting not only joints but bones and soft tissues as well [1, 2]. The condition is known to occur as a consequence of various peripheral neuropathies; however, by far, diabetic neuropathy is the most common [2, 3]. In its early stages, it is characterized by local inflammation, which usually progresses to various degrees of joint destruction, collapse, subluxation and deformities, and soft tissue affection, mainly ulceration [1, 4].

Various management options were suggested for dealing with foot and ankle CN, usually determined based on the pathology extension and degree of affection [4, 5]. In early disease stages, conservative management lines, including special footwear or total contact cast (TCC), are satisfactory [2, 6].

However, suppose the disease progresses and there is joint subluxation and deformity, with or without skin ulceration. In that case, various surgical interventions (including soft tissue and bony procedures) will be appropriate to achieve and maintain a stable plantigrade foot and ankle [7–9]. Furthermore, although severely complicated and infected CN cases could be candidates for amputation, various authors suggested that joint arthrodesis could be a valuable salvage option with a high incidence of avoiding amputations reaching between 80 and 100% [10–12].

During various arthrodesis procedures, surgeons could face bad local bone quality and/or bone defects necessitating reconstruction or augmentation to ensure proper union, which is usually carried out using bone grafts [13, 14], including autologous bone grafts (such as iliac bone grafts), allografts, and synthetic grafts; however, it carries the risk of donor site morbidity, disease transmission, and increased cost or unavailability for each grafts option respectively [14–16].

So, the primary objective was to report the union rate after only utilizing a locally obtained autologous bone graft while correcting the deformity and aiming for joint arthrodesis in patients with foot and ankle Charcot’s Neuroarthropathy. The secondary objectives were reporting the radiographic parameters (deformity correction), functional outcomes, and the incidence of the complications after a minimum of two years of follow up.

## Patients and methods

After obtaining the approval of our ethical committee (IRB no.: 17100867), we retrospectively looked at patients diagnosed with CN of the foot, ankle, or both who were admitted to our foot and ankle surgery unit (Affiliated with a North African Orthoapedic and traumatology department, in a tertiary university hospital) in the period between January 2016 to December 2018. The study was reported according to the PROCESS Guideline [17].

We included skeletally mature patients (> 18 years old) who were diagnosed with CN due to diabetic polyneuropathy (as proven by blood sugar levels, HBA1C, and nerve conduction velocity (NCV) studies), developed or impending ulcer formation, who were treated surgically due to unsatisfactory results of conservative treatment regarding and a follow up equal or more than 24 months. In contrast, patients who refused to participate in research activities, had incomplete records, had inadequate follow up, and those who presented with resistant infection or ischemia, which required primary amputations, were excluded. Thirty-seven patients met the inclusion criteria. Seven died before completing the required follow up period, and six were lost during follow up, leaving 24 patients for the final inclusion.

## Preoperative assessment

Clinical: History of any comorbidities (DM, HTN, etc.), generalized assessment as part of the preoperative preparation, including lower limb vascular status evaluation. Local foot and ankle evaluation was performed to assess the deformity (location, direction, and correctability), stability of the joints, walking pattern, and skin condition, including the site of ulceration, which, if present, further investigation was performed to rule out active infection (laboratory investigations including ESR and CRP, and if the ulcer was deep, a Probe to Bone test under aseptic conditions was performed to detect osteomyelitis and doing culture and sensitivity test).

Radiological: Weight-bearing plain radiographs include ankle anteroposterior (AP) and lateral views, while AP and oblique views were obtained for the foot. The location of affection, amount, and direction of the deformity were reported. The deformities were classified according to Brodsky’s classification [5, 18].

### Operative details

All patients were operated under spinal anesthesia in a supine position with the application of an above-knee tourniquet. In general, surgical intervention combines soft tissue releases (if necessary), fracture reduction (if required), and arthrodesis of the affected joints (as predetermined in the radiographs and the intraoperative evaluation).

Soft tissue releases: Achilles tendon Z-plasty aims to correct the equinus deformity and reduce the forces needed to be applied to correct the midfoot breakage. Then, fibrous tissue from the site of joint erosion was resected down to healthy bony tissue.

Bony procedures: We started with a preliminary trial of realigning any existing deformities and reducing fractures and/or dislocations in the ankle, subtalar joints, or midfoot. During this procedure, bone resection was performed if needed (any resected bone was kept as an autograft) to minimize the required forces for realignment and reduction; furthermore, it helped reduce the tension of the soft tissue envelope to minimize wound healing complications.

**Fig. 1:**
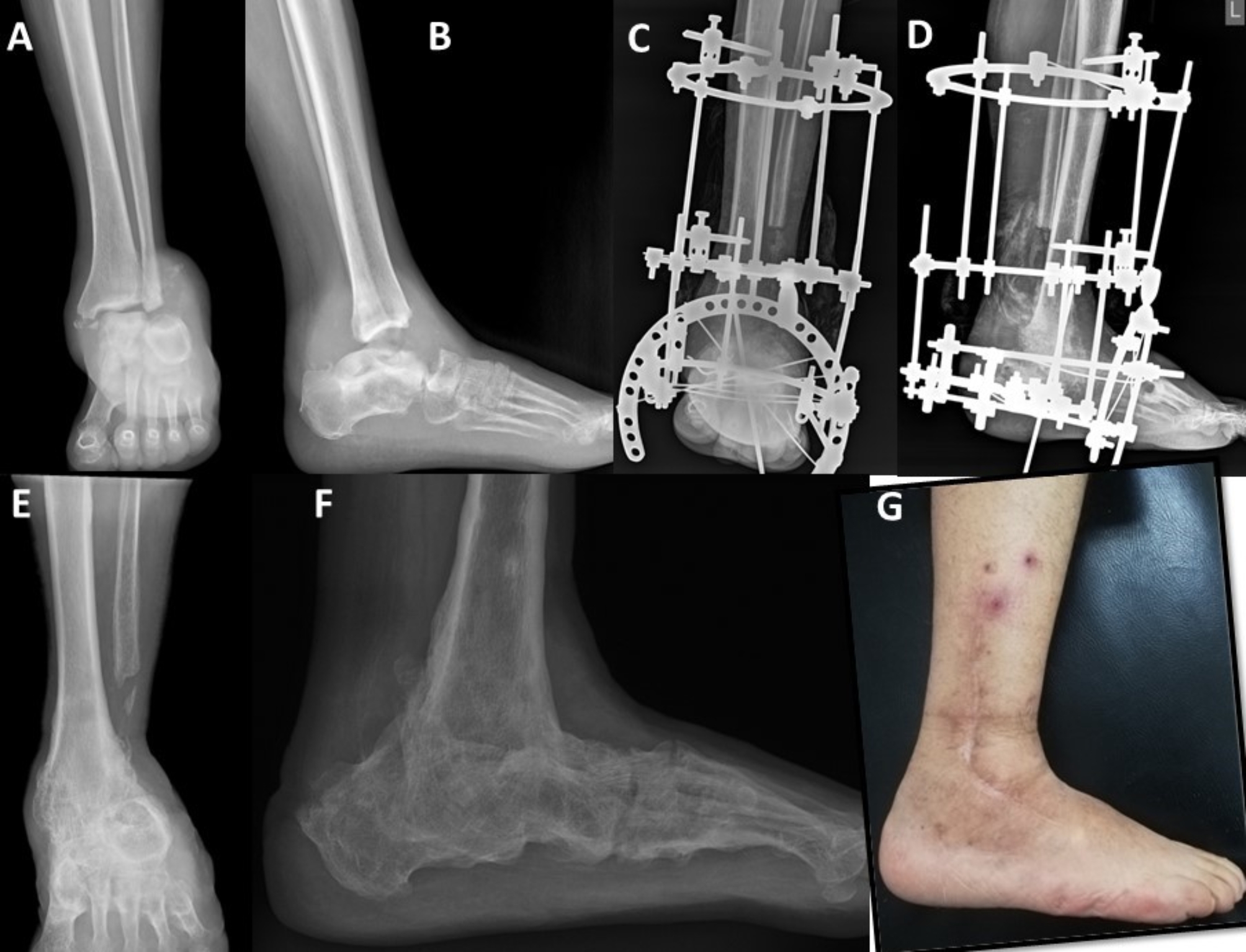
Male patient, 61 years old, diagnosed with CN of Brodsky type 3 A; he had an ulcer on the hindfoot. A and B, preoperative anteroposterior and lateral plain radiographs showing erosion of the talus and distal fibula. C and D Hindfoot fusion by Ilizarov external fixator was performed through a transfibular approach. A talectomy was performed, and the osteotomized distal fibula was used as a graft at the fusion site. E and F, radiological follow up after 30 months showing the solid bony union of the tibial and calcaneal articular surfaces. H, a clinical image showing healing of the ulcer and a plantigrade foot

**Fig. 2:**
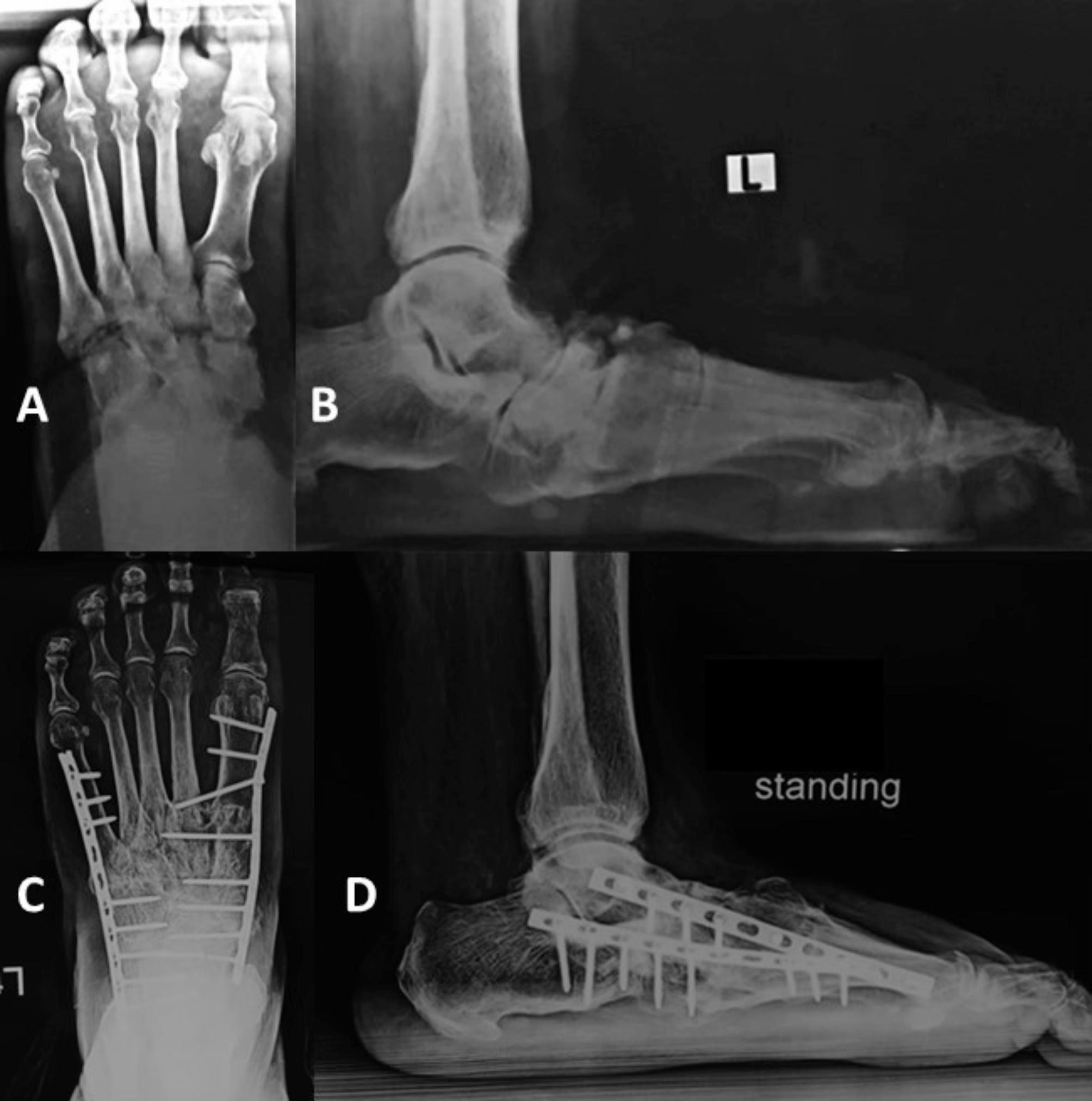
Female patient, 56 years old, diagnosed with midfoot CN with midfoot breakage. A, and B, preoperative anteroposterior and lateral plain radiographs. C and D, after 28 months of having a bi-columnar arthrodesis using plates and screws, there is a solid union of the arthrodesis sites

After obtaining satisfactory reduction and alignment, the arthrodesis surface was prepared by removing articular cartilage, then preliminary fixation using K-wires was performed, and the final position was checked under the image intensifier. Then definitive fixation depending on the skin condition was carried out as follows, Ilizarov external fixator in the presence of ulcers and internal fixation using either a calcaneal-tibial nail or plates and screws or a combination of both in cases with healthy skin (Figs. 1 and 2).

Bone grafting: None of our cases needed iliac bone autografts, allografts, or synthetic grafts. Only local autologous bone grafts, which were obtained from the resected distal part of the fibula or the resected part of the cuboid, navicular bone, or both in case of ankle or midfoot fusion, respectively, when needed. An additional graft was obtained from the distal tibia surface, which was not in contact with the talus or calcaneus in case of hindfoot fusion. The obtained bone was morselized into 2–3 mm chips mixed with Vancomycin antibiotic powder and was impacted in the areas with bone defects. Tension-free wound closure was performed after insertion of a negative suction drain.

### Postoperative care

Intravenous antibiotics were administered during the hospital stay and converted to oral antibiotics after discharge until the sutures were removed at two weeks. Deep venous thrombosis prophylaxis was prescribed for at least four weeks or until the patient started active weight bearing.

Regardless of the fixation method, all patients had a non-weight-bearing protocol for the first six weeks. Furthermore, the decision to start gradual weight-bearing was made individually based on the progression of the arthrodesis site union monitored in plain radiographs.

In the internal fixation groups, a below-knee slab was applied till wound healing and removal of the sutures; then, it was replaced by a below-knee cast. When an external fixator was used, there was a gradual increase in weight bearing until full weight bearing was achieved. For frame removal, it was dynamized by loosening the rods connecting the hindfoot to the leg and those connecting the hindfoot to the foot for six weeks. The loosened rods were sequentially removed every two weeks till the complete removal of all rods and, hence, the frame. By this protocol, no cast was needed. After cast or external fixator removal, weight-bearing in a Controlled Ankle Motion Walker (CAM-Walker) was continued for at least one year; all patients were advised to use on-the-shelf diabetic foot shoes.

### Assessment during follow up visits

Patients were followed up in the clinic at one week for wound evaluation and two weeks for suture removal. Starting from six weeks postoperatively and then monthly, the same set of plain radiographs obtained preoperatively were performed to assess the union of the fusion surface, loosen the implants, and maintain reduction and alignment. After ensuring complete union, patients were followed every two months, and after the first postoperative year, patients were advised to visit the clinic every six months.

*The following assessments were performed*,* and the results obtained by the last follow up were reported*:

### Radiographic outcomes

- Deformity correction was evaluated in plain radiographs by assessing the following parameters: for patients who had midfoot bi-columnar fusion, AP and lateral Meary´s angles (talo- first metatarsal angle), Hibb´s angle (calcaneo-first metatarsal angle), Calcaneal pitch angle, and Forefoot adducts, while for patients who had Hindfoot fusion, Calcaneal pitch angle, and Tibio-calcaneal angle were measured [19].
- To assess the union, we relied on clinical and radiological criteria: Clinically, if there was no swelling, painless weight bearing, and no gross instability on manipulation. While the radiological union was decided if there was bony bridging on both radiographic views, trabecular bridging across the fusion sites, obliteration of the arthrodesis surface, and no progression of the deformity in follow up serial radiographs [7, 20–22]. Non-union was defined if the above criteria were not achieved after six months postoperatively.

### Clinical outcomes

- The function was assessed using a modified American Orthopaedic Foot and Ankle Society ankle hindfoot scale (mAOFAS); after the deduction of 14 points representing the ankle and subtalar movement, the final score had 86 points instead of 100 points of the original scale, excellent results if the score was 86 to 74, good if 73 to 64, fair if 63 to 54, and poor if less than 54 [23, 24].
- Ulcer healing was assessed using the revised photographic wound assessment tool (revPWAT) [25].
- Patient satisfaction was assessed by asking two simple questions, and the answers were yes or no: 1-if the patient was satisfied by the final functional results, and 2-if the patient would recommend the surgery for another patient with the same condition.

Complications occurring perioperatively or during the follow up period were reported.

### Statistical analysis

The collected data were analyzed using SPSS (Statistical Package for the Social Sciences, version 20, IBM, Armonk, New York). The Shapiro-Wilk test was used to determine whether the data was normally distributed. Data were presented as median (minimum-maximum) for non-normally distributed data, mean ± standard deviation for normally distributed data, and number (n) and percentage (%) for nominal data. Data that did not follow a normal distribution were compared using the Wilcoxon signed-rank or Mann-Whitney U tests, depending on the data context. A P-value of less than 0.05 was considered statistically significant.

## Results

The included patients had a mean age of 55.4 ± 10.1 (30–71) years, 13 (54.5%) were males, and all included patients had unilateral lower limb affection (15 (62.5%) right side and 9 (37.5%) left side). Seven (29.2%) cases were classified as Brodsky type 1, 11 (45.8%) as type 3 A, and six (25%) were type 4. Details of the arthrodesis anatomical location and the fixation methods used are presented in Table 1. Eight (33.3%) cases had preoperative ulcers, located in the heel region in three, on the lateral midfoot in three, on the medial midfoot in one, and on the lateral aspect of the ankle in one. There was no significant difference regarding the presence of ulcers in relation to the Brodsky classification (Table 2). All patients were available till a mean follow up of 35.7 ± 9.5 (24–54) months.

**Table 1:** Procedural and fixation methods among studied patients

Anatomical location	Hindfoot fusion Tibio-Talo-Calcaneal fusion Pantalar arthrodesis Ankle arthrodesis	17 (70.8%) 13 (54.2%) 3 (12.5%) 1 (4.2%)
	Midfoot bi-columnar fusion	7 (29.2%)
**Fixation methods**	Ilizarov external fixator	15 (62.5%)
	plates and screws*	7 (29.2%)
	calcaneal-tibial nail	2 (8.3%)

*One patient from this group developed an infection, and the plate and screws were removed and replaced by Ilizarov external fixator

**Table 2:** Ulcer among the studied group based on Brodsky types (preoperatively):

Presence of ulcer	Brodsky type*		
	Type 1 (7 feet)	Type 3 A (11 feet)	Type 4 (6 feet)
No	4 (57.1%)	8 (36.4%)	4 (66.7%)
Yes	3 (42.9%)	3 (27.3%)	2 (33.3%)
*P* value**	0.79		

*Data expressed as frequency (percentage). **Data was compared using the Mann-Whitney U test.

### Radiographic outcomes

arthrodesis site union was achieved in 23 (95.8%) cases after a mean of 4 ± 1.7 (2-7.5) months. One (4.2%) case, classified as Brodsky type 3 A, failed to achieve union, which was advised for revision, but the patient refused.

All the measured radiographic parameters significantly improved at the last follow-up assessment compared to the preoperative values except for the Hibb´s angle (Table 3).

**Table 3:** Radiographic correction of the deformities

	Preoperatively*	Last follow up*	*P* value**
**bicolumnar fusion group**			
Meary´s angle (°)	-26 (-37 to -20)	-5 (-28 to 0)	**0.01**
Hibb´s angle (°)	164 (-13 to 169)	150 (141 to 160)	0.23
Calcaneal pitch angle (°)	8 (-2 to10)	10 (3 to 25)	**0.02**
Fore foot adductus (°)	-5 (-17 to 4)	9 (2/11 to 30)	**0.01**
Talo 1st metatarsal angle (°)	-13 (-20 to 0)	1 (-1 to 12)	**0.02**
**hindfoot fusion group**			
Calcaneal pitch angle (°)	10 (-18 to 30)	19 (-9 to 24)	**< 0.05**
Tibio-calcaneal angle (°)	85 (63 to 116)	74 (47 to 100)	**< 0.05**

*Data expressed as median (range). **Data was compared using the Wilcoxon test.

### Clinical outcomes

Their functional outcomes at the last follow up had a mean mAOFAS of 72.4 ± 10.41 (46–83) points, and the mean subscales score were as follows, 35.38 ± 5.04 (30–40) for pain, 28.4 ± 9.39 (28–42) for function, and 9.41 ± 2.08 (8–10) for alignment, 19 (79.2%) patients achieved excellent and good scores, while five (20.8%) reported fair or poor scores. According to the revPWAT score, seven (87.5%) patients had healed ulcers during the follow-up. The unhealed one was due to poor blood sugar control with CN reactivation and ankle fusion nonunion. Twenty-two (91.7%) patients were satisfied with their functional results, and 19 (79.2%) patients would recommend the same surgery for patients with similar conditions.

### Complications

Infection was encountered in three (12.5%) cases managed by debridement and antibiotics, and removing the plate and screws and refixation using an Ilizarov external fixator was needed in one of them. Tibia fracture at the site of previous pin-tract infection occurred in two (8.3%) cases who received local debridement and above knee cast till fracture union was achieved in both. A delayed union of the fusion site was encountered in one (4.1%) patient with a calcaneal-tibial nail and the union was achieved two months after nail dynamizations. One patient (4.1%) developed late postoperative (at three weeks) bleeding from a pin-tract site due to the development of an aneurysm of the anterior tibial artery. It was managed by emergency arterial embolization with no further consequences.

## Discussion

Union rates after foot and ankle joint arthrodesis performed for managing various pathologies, including CN, have been reported in the literature to range from 64 to 100% [7, 22, 26–29]. However, risk factors leading to delayed or non-union have been studied, including fixation techniques and local foot and ankle bone quality [26, 30].

In a comparative study by La Fontaine et al., the authors compared the bone quality in normal individuals and those having foot and ankle CN; they found that the histologic examination of CN showed inflammation, myxoid infiltrates, and disorganized trabecular pattern compared to normal bone, they concluded that bone in CN is fragile and has impaired repair process [31]. Furthermore, some cases present with significant bone defects or defects resulting after joint debridement and preparation [8, 13]. Owing to the previously mentioned reasons, there is a need for bone healing stimulators in the form of bone grafts to ensure proper union at the arthrodesis sites and to reconstruct bone defects if present when operating on foot and ankle CN [13–15, 32, 33].

In the current series, after implementing only locally obtained bone autografts, we obtained a union rate of about 96% in patients who underwent different joint arthrodesis to manage foot and ankle CN. The deformity correction was maintained till the last follow up with acceptable functional and patient satisfaction outcomes.

Which joint to be fused while managing foot and ankle CN and whether soft tissue procedures are needed are dependent on the degree of joint affection and the need for deformity correction; furthermore, obtaining stable joints is mandatory for soft tissue envelope and ulcer healing [8–10]. In the current study, we had various joints affection (Type 1, 3 A, and 4 according to Brodsky classification); accordingly, different joints (hindfoot and midtarsal) needed to be fused. The same variability of joint arthrodesis was reported in previous studies [7, 12]. Consequently, fixation tools vary greatly; these could be selected based on the surgeon’s preference; however, considering the status of the soft tissue envelope, the presence of infection or ulcers is crucial for selecting the proper fixation tool [7–9, 23].

Bone graft options include autografts (including locally obtained grafts, iliac bone grafts, and femoral reamer-irrigator aspirator (RIA) bone grafts) [14, 34–36], allografts, bone graft substitutes (such as demineralized bone matrix, calcium sulfate, calcium phosphate, and tricalcium hydroxyapatite), and more recently, biologic agents were introduced, including recombinant human bone morphogenetic protein-2 or recombinant human platelet-derived growth factors [32, 33, 37, 38]. These graft types usually differ concerning their osteoinductive, osteoconductive, osteogenic, and angiogenetic properties; however, autografts are the benchmark graft for foot and ankle arthrodesis, as they combine the previously mentioned four properties [15, 32, 33].

The efficacy of locally obtained autografts for obtaining arthrodesis during foot and ankle surgeries has been reported in the literature [7, 13, 16, 36]. In the current series, we only used local autografts obtained from the distal fibula (as part of the trans-fibular approach), the resected bone (cuboid and navicular), or the excess bone removed during debridement. Another source for local graft was the distal part of the tibia, which was not articulating with the talus. We achieved arthrodesis site union in nearly all cases; furthermore, we avoided the possible donor site morbidity, chronic graft donor site pain, and longer operative time associated with obtaining iliac bone grafts and the extra cost and possible disease transmission associated with synthetic grafts and allografts, respectively [14, 15].

In a study by Rana and Patel, including 46 operated anatomical locations in 44 patients, the authors reported using the excised fibula as a local autograft to reconstruct severely defective talus in 15 Tibiotalocalcaneal (TTC) fusion and two Pantalar fusion; however, they enhanced the local healing environment by adding iliac bone cortico-cancellous grafts; furthermore, the authors mentioned using only the resected fibula in some cases, leaving its posterior soft tissue envelope attached to act as a local vascularized autograft to assist in arthrodesis site union [7].

In the current series, we achieved an arthrodesis site union in 95.8% of the patients at a mean of 4 ± 1.7 months and a non-union rate of 4.2%. Similar to our results, Ersin et al. achieved union in 23 out of 24 CN patients who underwent hindfoot arthrodesis using retrograde intramedullary nailing after a mean of 10 months [29]. However, lower union rates were reported, where Rana and Patel reported primary union in 65% of any of the included anatomical locations at a mean follow up of 6.8 months [7]. Furthermore, non-union rates of 34% and 27% were reported by Papa et al. [27] and Schwartz et al. [28], respectively. The differences in the union rates among studies could be attributed to the different anatomical locations in each study, different fixation methods, and variability in patient-related factors.

The patients included in our series achieved a mean mAOFAS of 72.4 ± 10.41 (46–83) points, which was similar to the results obtained by ElAlfy et al. who treated 27 patients with foot and ankle CN (14 had Illizarov while 13 had IMN), the authors measured their functional outcomes using the same score used in the current series, they reported a mean score of 80 ± 2.7 and 75 ± 1.9 points for each fixation group respectively [23]. Furthermore, 77.8% of their patients achieved either excellent or good outcomes; we achieved similar results, whereas 79.2% of our patients achieved the same outcomes.

Eleven patients in ElAlfy et al. had skin ulcers (7 were on the lateral aspect of the ankle and 4 on the medial side); they reported that all ulcers had healed by a mean follow up of seven weeks [23]. In the current series, all but one ulcer had healed, attributed to poor blood glucose control. We believe optimum blood glucose levels, proper deformity correction (to prevent undue pressure areas), and solid union at arthrodesis sites are paramount contributors to ulcer healing.

The commonest complication in the current series was an infection, which occurred at an incidence of 12.5%, necessitating debridement in all and hardware removal in one patient, and no amputation was required in any patient. A slightly higher rate of 16.7% was reported in the Ersin et al. study; however, all were treated with local debridement and antibiotics, and no patient required amputation [29]. Unfortunately, in foot and ankle CN patients, complications could be devastating, Papa et al. [27] and Schwarz et al. [28] reported complications incidence in 65% and 53% of their patients, respectively, most of them were infections. Rana and Patel reported an infection incidence of 13%; however, one of their patients with resistant polymicrobial infection required below-knee amputation [7].

Our study has several limitations. First, being a retrospective, no comparative study, it carried a higher risk of recall and selection biases. Second, the relatively small number of the included patients which was further affected by patients’ deaths or those lost during follow up. Third, we could not obtain the preoperative functional scores values from records and reported only the last follow up scores without comparison, which was attributed to the difference in the score used. Last, arthrodesis consolidation and union were assessed using plain radiographs, which are inferior to CT scans as reported in the literature [21, 22].

## Conclusion

Foot and ankle Charcot neuroarthropathy is a demanding condition, which, if inappropriately managed, could lead to devastating complications up to limb amputation. Deformity correction by arthrodesis of the affected joint is a valid management option, resulting in acceptable clinical and radiological outcomes. To enhance the local environment for arthrodesis consolidation, using locally obtained autografts seems valid and convenient, leading to higher union rates and avoiding drawbacks associated with using other graft types.

## Data Availability

No datasets were generated or analysed during the current study.

## Declarations

### Study setting

Foot and Ankle Surgery unit, Orthopaedic Department, Assiut University Hospital, Assiut, Egypt.
